# Mutualistic strategies minimize coextinction in plant–disperser networks

**DOI:** 10.1098/rspb.2016.2302

**Published:** 2017-05-10

**Authors:** Evan C. Fricke, Joshua J. Tewksbury, Elizabeth M. Wandrag, Haldre S. Rogers

**Affiliations:** 1 Department of Ecology, Evolution, and Organismal Biology, Iowa State University, Ames, IA 50011, USA; 2 Colorado Global Hub, Future Earth, Boulder, CO 80309, USA; 3 Sustainability, Energy and Environment Complex, University of Colorado, Boulder, CO 80309, USA; 4 School of Global Environmental Studies, Colorado State University, Fort Collins, CO 80523, USA; 5 Institute for Applied Ecology, University of Canberra, Canberra, Australian Capital Territory 2617, Australia

**Keywords:** defaunation, ecological networks, global change, mutualism, plant–animal interactions, seed dispersal

## Abstract

The global decline of mutualists such as pollinators and seed dispersers may cause negative direct and indirect impacts on biodiversity. Mutualistic network models used to understand the stability of mutualistic systems indicate that species with low partner diversity are most vulnerable to coextinction following mutualism disruption. However, existing models have not considered how species vary in their dependence on mutualistic interactions for reproduction or survival, overlooking the potential influence of this variation on species' coextinction vulnerability and on network stability. Using global databases and field experiments focused on the seed dispersal mutualism, we found that plants and animals that depend heavily on mutualistic interactions have higher partner diversity. Under simulated network disruption, this empirical relationship strongly reduced coextinction because the species most likely to lose mutualists depend least on their mutualists. The pattern also reduced the importance of network structure for stability; nested network structure had little effect on coextinction after simulations incorporated the empirically derived relationship between partner diversity and mutualistic dependence. Our results highlight a previously unknown source of stability in mutualistic networks and suggest that differences among species in their mutualistic strategy, rather than network structure, primarily accounts for stability in mutualistic communities.

## 1. Introduction

The current rates of anthropogenic extinction are unprecedented [1], but the resulting extinction of ecological interactions may cause far more pervasive impacts [2], including widespread coextinction of dependent species such as mutualists [3]. Our understanding of the traits and circumstances that predict coextinction risk following mutualism disruption is still in its infancy [4–6]. Research has focused on the number and identity of partners as determinants of coextinction risk, predicting that coextinction risk decreases with greater partner diversity [6–9]. Species with many partners can be rescued by their remaining partners when one partner is lost from the community, whereas a species with a single partner cannot. Network models that simulate mutualism disruption confirm that species with few partners are more likely to experience mutualism loss and thus experience coextinction [7,10–13].

Another determinant of coextinction risk that has been less well studied is the degree to which species depend on a particular type of mutualism for reproduction or survival, or their ‘mutualistic dependence’ [14]. Species that participate in mutualisms vary widely in mutualistic dependence, with many species adopting partially mutualistic strategies in which the mutualism is beneficial but not obligatory [15–17]. For example, plants whose seeds get dispersed by animals vary in the necessity of animal dispersal for regeneration, and animals that pollinate plants vary in the importance of floral rewards in their diets. A species with one partner—despite lacking redundancy in mutualistic partners and, as a result, having high risk of mutualism loss—could have low coextinction risk if it is an opportunistic mutualist with low mutualistic dependence. Thus mutualistic dependence and partner diversity should collectively determine species’ responses to mutualism disruption.

Ecological theory developed independent of network research [16] and some empirical data [18–20] suggest that there is a positive relationship between mutualistic partner diversity and mutualistic dependence. Species with high mutualistic dependence are expected to interact with many partners to avoid risks caused by species-specific fluctuations in mutualistic resources or services [16], whereas the costs of maintaining mutualistic interactions [21] likely limit species with low mutualistic dependence from maintaining interactions with many partners. Empirical studies that categorize animals in seed dispersal networks as obligate, partial, or opportunistic frugivores and report their plant partner diversity [19,20,22] suggest that mutualistic dependence is positively related to partner diversity. One study that has assessed mutualistic dependence of animal-pollinated plants also supports this relationship; plants with fewer mutualists were more likely to set seed when animal pollinators were excluded, indicating lower mutualistic dependence [18]. Importantly, a positive relationship between partner diversity and mutualistic dependence should reduce coextinction because the species most likely to lose mutualistic interactions would be those that can best persist in their absence. Likewise, species that depend heavily on mutualistic interactions would be unlikely to experience coextinction because they possess redundant mutualists. Although the few studies that assess the relationship between mutualistic dependence and partner diversity suggest the generality of this positive relationship [18–20], none have considered that the relationship may confer stability to mutualistic networks.

Mutualistic network models have examined the importance of patterns in the diversity and identity of partners—network structure—for coextinction, assessing implications for stability and coexistence [7,11,13,23], but have not included empirical information on mutualistic dependence. Instead, current network models assume that there is no systematic variation in mutualistic dependence among species [14], with all species depending entirely on the mutualism for reproduction and survival [7,10,24–27] or with mutualistic dependence varying randomly with respect to partner diversity [11]. Thus the models used to date to explore coextinction and mutualistic community dynamics have assumed that all species that participate in mutualisms are similarly and heavily dependent on mutualistic interactions.

The assumption that all species depend heavily on mutualistic interactions underlies the putative links between partner diversity and coextinction risk and between network structure and stability. Because species with few mutualists are more likely to lose all of their partners after network perturbations, the assumption of equal mutualistic dependence leads to the prediction that species with low partner diversity are more vulnerable to coextinction. The link between network structure and stability in turn results from this relationship between partner diversity and coextinction risk [28]. Because in nature the species considered most vulnerable to mutualist loss (those with few partners) tend to interact with partners considered to have lowest extinction risk (those with many partners), empirical network structure is thought to minimize coextinction and therefore confer stability [9,11,29]. Thus in the absence of information on mutualistic dependence, network structure appears to be critical for reducing coextinction and favouring stability. However, in models that allow variation in mutualistic dependence, any positive relationship between partner diversity and mutualistic dependence would weaken the relationship between partner diversity and coextinction risk and, in turn, diminish the influence of network structure on stability. In other words, if systematic variation in mutualistic dependence mediates network robustness such that coextinction risk is typically low and weakly related to partner diversity, a species with a single mutualist would not derive the same benefit from interacting with a species that has many partners over a species that has few partners. A positive relationship between partner diversity and mutualistic dependence should therefore reduce the importance of network structure for stability.

We tested empirically the relationship between species' mutualistic partner diversity and their dependence on mutualism, then used simulations to assess the influence of this relationship on the behaviour of mutualistic networks undergoing mutualism disruption. We focus on plant–animal seed dispersal mutualism and report two empirical tests using 30 networks that together include 419 animal and 808 plant taxa. First, we use global interaction network and diet databases to assess the relationship between the diversity of animals' plant partners and their dietary dependence on fruit in 29 globally distributed seed dispersal networks. Second, we pair detailed field experiments and observations to assess the relationship between the diversity of plants' mutualistic animal partners and the magnitude of the benefits that they receive from seed dispersal. We rely on a local test because a global test of the dependence of fruiting plants on their frugivores is limited by a lack of plant demographic data [6]. Finally, we incorporate our empirical findings into a network model to assess the effect of the relationship on coextinction. We use a stochastic simulation model [14] that allows inclusion of data on mutualistic dependence and quantitative interaction data. Under existing assumptions, this model yields qualitatively equivalent conclusions regarding the importance of partner diversity and network structure for extinction as the other topological (e.g. [7]) and dynamical (e.g. [11]) models described above. By simulating extinction in the presence or absence of empirical network structure and the empirical relationship between partner diversity and mutualistic dependence, we compare the relative importance of each of these factors for minimizing coextinction in mutualistic networks.

## 2. Methods

### (a) Dependence on frugivory in global seed dispersal networks

We examined the relationship between partner diversity and the degree of frugivory among animals in a globally distributed set of 29 empirical seed dispersal networks (available at www.web-of-life.es; see electronic supplementary material, table S1 for references and network description). These networks report species-specific data on observed interactions. We separately considered the 11 quantitative networks, where connections weighted by interaction frequency enable strong inference [25], and the 18 binary networks, where only the presence or absence of interaction was recorded. These networks are commonly included in mutualistic network studies that focus on network structure and its implications for coextinction and stability [11,13,24]. We calculated several network metrics to describe partner diversity for use in the analysis of the quantitative networks. Species degree is the number of partners with which the focal species interacts. Species strength is a quantitative equivalent of species degree calculated by taking the sum, across all partner species, of the portion of the partner species' interactions that are with the focal species [8]. Total interaction frequency was calculated as the sum, across all partner species, of interaction frequency with the focal species. We also calculated the Shannon diversity (H) of interaction frequency. For the binary networks we used only species degree to describe partner diversity. To characterize the animal species' dependence on fruit, we used estimates of the portion of the animal's diet comprised of fruit from EltonTraits 1.0 [30]. In this database, the portion of the species’ diet that is fruit is estimated to the nearest 10%, which in the analysis we treated as a score from 0 to 10. In separate generalized linear mixed effects models with each network metric as the fixed effect, the binomial response variable was the portion of diet that is fruit and we allowed random slopes and intercepts by network ID. To determine statistical significance, we used likelihood ratio tests to compare the models described above to intercept-only models lacking the fixed effect.

### (b) Dependence on seed dispersal in Mariana Island fruit–frugivore network

To determine how the dependence of plants on their frugivore partners is related to the diversity of their animal partners, we studied interactions between trees and seed dispersers in the Mariana Island chain of the western Pacific Ocean. The islands have a short forest canopy with low species richness and receive 2.0–2.5 m of rain annually with a dry season from January to June. Observations and experiments were conducted across the inhabited Mariana Islands of Guam, Rota, Tinian and Saipan. We developed a seed dispersal network on the island of Saipan, which possesses the most intact assemblage of native frugivores among the inhabited Mariana Islands.

We measured benefits of seed dispersal for seeds and seedlings with sets of experiments that focused on a particular benefit of dispersal at the seed or seedling stage. These manipulative experiments assessed benefits associated with escape from distance-dependent mortality, movement to high light areas, and handling of fruits by frugivores [31,32] (see electronic supplementary material, appendix S1 for detailed methods). To quantify the degree to which plant individuals benefit from dispersal, we calculated the ratio of survival in a ‘dispersed’ scenario (far from conspecifics, in high light areas, seeds handled by frugivores) versus in a ‘non-dispersed’ scenario (near conspecifics, under closed canopy, whole fruit unhandled by frugivores), and report this as the ‘dispersal benefit ratio’ (electronic supplementary material, appendix S1).

We developed a seed dispersal network using observations of frugivores visiting focal tree species on Saipan from May to August in 2013 and 2014. These months encompass the beginning of the wet season and the time of peak fruiting. Observations were conducted in three forest sites across the island. We recorded frugivory through extended direct observation of focal fruiting trees [22], using an average of 220 h of observation per plant species to develop the quantitative network (see electronic supplementary material, appendix S1 for detailed methods).

To determine whether plants that benefit more from dispersal also have more partners, we used linear mixed effects models with the dispersal benefit ratio from the experiments described above as the response variable and each quantitative network metric describing partner diversity as the single fixed effect. By allowing random slopes and intercepts by experiment ID (e.g. seed stage distance dependence experiment, seedling stage distance dependence experiment), we assess the overall relationship between partner diversity and the dispersal benefit ratio across all experiments. Using this approach, the presence of a positive effect of partner diversity would indicate that, even with variation among experiments in the magnitude of the benefit or slope of the relationship, species with greater partner diversity have greater benefits of dispersal. To assess statistical significance, we use likelihood ratio tests to compare these models to a null model lacking a fixed effect.

### (c) The influence of mutualistic dependence on coextinction predictions

We tested the influence of a relationship between partner diversity and mutualistic dependence on the robustness of networks to coextinction by predicting coextinctions within the 11 quantitative seed dispersal networks. These networks allowed us to simulate extinctions using networks possessing empirical variation in network properties. The networks do not provide information on species’ mutualistic dependence, and we assigned mutualistic dependence to species as described below.

We used a stochastic coextinction model developed by Vieira & Almeida Neto [14]. Other models have also been used to assess coextinction and stability in mutualistic networks. Topological models [7,27] incorporate only binary interaction data and do not allow inclusion of information on variation among species in mutualistic dependence; species simply experience extinction when their last remaining partner experiences extinction. Dynamical models, typically using Lotka–Volterra equations and binary interaction data (e.g. [11]), allow variation among species via differences in parameters including intrinsic growth rate, competition coefficients and mutualistic coefficients. Obtaining such parameters empirically in complex communities is logistically unfeasible [33], especially for the long-lived plants and frugivores that are the focus of this study. The stochastic simulation we use allows inclusion of quantitative interaction data and includes a term describing the dependence of each species on the mutualism as a whole. This stochastic simulation model therefore offers an empirically tractable approach for incorporating empirical variation in mutualistic dependence.

In this model, the probability of coextinction of each species is given by the portion of a focal species' interactions that were observed to be with the now-extinct mutualist multiplied by the mutualistic dependence of the focal species on the mutualism as a whole (*R_i_*, values 0–1). Each iteration of the simulation begins with the extinction of a randomly chosen species. Coextinctions are allowed to occur as the result of the initial extinction, and then coextinctions of progressively higher order occur (e.g. secondary coextinctions that result from primary coextinctions, etc.) until no further extinctions are observed. The number of coextinctions is recorded, and another iteration of the simulation begins.

We modified the approach developed by Vieira & Almeida Neto [14] to allow variation between species in their mutualistic dependence as a function of species strength. To assess how the observed relationship between partner diversity and mutualistic dependence influences coextinction, we performed simulations within the 11 seed dispersal networks under two scenarios. In the ‘obligate scenario’, all species were assumed to be obligate mutualists (*R_i_* = 1 for all species). In the ‘observed scenario’, the relationship between species strength and mutualistic dependence for both plants and animals was given by the mean relationship between species strength and the degree of frugivory exhibited by animals in the 11 quantitative seed dispersal networks. To assess the influence of any non-random attributes of network structure on coextinction, we compared empirical networks, possessing any of these attributes (e.g. nestedness, compartmentalization), to the same networks after they were randomized. The randomization approach we used maintains the total number of interaction events per species [34].

We performed simulations using either empirical or randomized networks and, for the relationship between partner diversity and mutualistic dependence, either the ‘obligate scenario’ or the ‘observed scenario’. For each of these four combinations, we recorded the portion of species that experienced coextinction in 10 000 iterations of the simulation for each network. To assess the relationship between partner diversity and vulnerability to coextinction, we recorded the species strength and the coextinction status (coextinct or not) for every individual in every fifth iteration. For data visualization, we plot model estimates from a generalized linear mixed effects model for the obligate scenario (under the assumption that all species are obligate mutualists) and one for the observed scenario (using the empirical relationship between species strength and degree of frugivory) with coextinction status as the response variable, species strength as the fixed effect, and random slopes and intercepts by network ID. We performed additional simulations, described in electronic supplementary material, appendix S1, to relax the assumption of obligate mutualisms and explore how other potential relationships between partner diversity and mutualistic dependence influence coextinction.

## 3. Results

### (a) Relationship between partner diversity and mutualistic dependence

**Figure 1.**
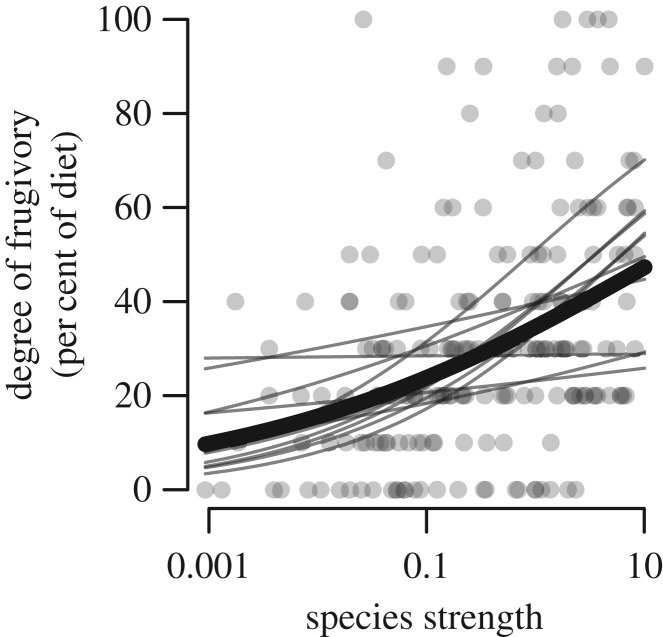
A positive relationship between partner diversity and mutualistic dependence among animals in 11 empirical quantitative seed dispersal networks. Thin lines represent model fits for each network and the thick line represents the mean model fit.

Our analyses of the mutualistic dependence of frugivores included 406 species of birds (51 families, 202 genera) and mammals (12 families, 26 genera) identified to the species level. In the 11 quantitative networks, we found positive relationships between all metrics describing partner diversity and the degree of frugivory (portion of diet that is fruit). Species strength, a quantitative network metric that combines information on the number of partners and frequency of interaction [8], was positively related to the degree of frugivory (*χ*^2^ = 10.3, d.f. = 1, *p* = 0.001; figure 1), as were other related network metrics including the number of mutualists (*χ*^2^ = 9.9, d.f. = 1, *p* = 0.002), total interaction frequency (*χ*^2^ = 7.2, d.f. = 1, *p* = 0.007), and Shannon diversity (*χ*^2^ = 8.6, d.f. = 1, *p* = 0.003). In the additional set of 18 binary networks, where only the presence or absence of an interaction is recorded, the number of mutualists was also positively related to the degree of frugivory (*χ*^2^ = 6.5, d.f. = 1, *p* = 0.011). Animals that interact with a higher diversity of fruiting plants depend more on the seed dispersal mutualism than do species with fewer mutualistic partners. In contrast, species with fewer partners were likely to be only partially or opportunistically frugivorous, depending primarily on other diet items.

**Figure 2.**
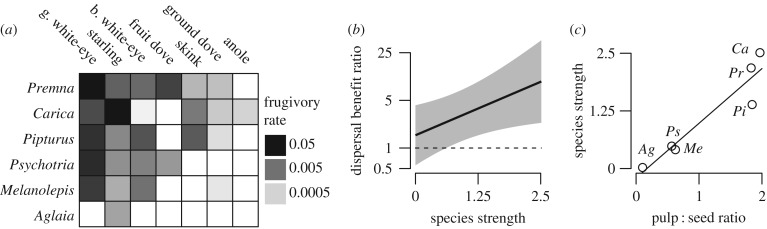
Fruit–frugivore interactions and the benefits of seed dispersal for plants in the Mariana Islands. (*a*) Seed dispersal network, with columns representing animal species and rows representing plant species, referred to by genus. (*b*) The relationship between species strength and the dispersal benefit ratio for plants (model output with 95% confidence intervals; detailed results in electronic supplementary material, figure S1). (*c*) Relationship between the pulp-to-seed ratio and species strength.

In our second test, we studied a network including seven frugivore species (five families) and six plant species (six families) to examine the dependence of fruiting plants on fruit-eating animals (interaction frequencies shown in figure 2*a*). Species strength was positively related to the dispersal benefit ratio (ratio of survival in a dispersed and in a non-dispersed scenario; *χ*^2^ = 4.01, d.f. = 1, *p* = 0.045; figure 2*b* and electronic supplementary material, figure S1), as were the other network metrics (all *p* < 0.05). We also found that mutualistic reward traits influence the identity of plant partners for frugivores; plant species that invested more reproductive mass toward mutualist attraction—by producing relatively more fruit pulp—had greater species strength (*F*_1,4_ = 33.04, *p* = 0.005; figure 2*c*). Plants with more frugivore partners were more dependent upon the mutualism for seed and seedling survival than those with fewer partners.

### (b) Coextinction predictions

**Figure 3.**
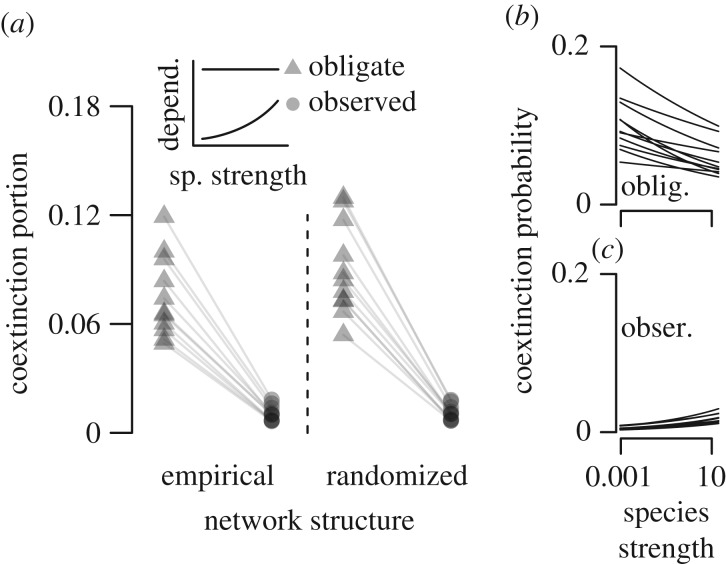
The positive relationship between partner diversity and mutualistic dependence reduces coextinction and alters the influence of network structure and partner diversity on coextinction. (*a*) The portion of species experiencing coextinction in simulations within 11 quantitative networks when assuming all species are obligate mutualists (triangles; horizontal line in inset panel) or using the observed relationship (circles; positive relationship in inset panel). Compare between randomized and empirical structure to assess the decrease in coextinction due to empirical network structure when assuming all species are obligate mutualists (compare triangles between randomized and empirical structure) or when using the observed relationship (compare circles). Simulated within the 11 empirical networks, the relationship between partner diversity and vulnerability to coextinction when assuming that all species are obligate mutualists (*b*) or when using the observed relationship (*c*).

The observed relationship between species strength and mutualistic dependence (measured as the animal species' degree of frugivory in quantitative networks; figure 1) reduced coextinction by 88% relative to coextinction predictions made under the assumption that all species are obligate mutualists (empirical structure scenario, average reduction, figure 3*a*). Additional simulations showed that positive relationships between partner diversity and mutualistic dependence consistently reduce coextinction and that this effect is not explained simply by a system-wide decrease in mutualistic dependence (electronic supplementary material, figure S2). Because species that participate in these networks typically possessed partially mutualistic strategies, and because species that were more likely to lose all of their partners also depended less on the mutualism, the mutualistic networks were highly robust to coextinction.

### (c) Importance of network structure

We next reconsidered the importance of network structure for stability in light of the observed relationship between partner diversity and mutualistic dependence. First, we assessed stability caused by empirical network structure while assuming that all species were obligate mutualists. We found that empirical networks had on average 17% fewer coextinctions than did randomized networks (figure 3*a*), indicating that empirical network structure confers stability when assuming all species are obligate mutualists. Also following existing predictions, coextinction was much more likely among species with low partner diversity (figure 3*b*).

Next, we included the observed relationship between partner diversity and mutualistic dependence and again simulated extinction in empirical and randomized networks. The importance of network structure for stability strongly decreased, with empirical networks producing only 3.6% fewer coextinctions than did randomized networks (figure 3*a*). Further, coextinction was not more likely among species with low partner diversity (figure 3*c*). Although including the observed relationship between partner diversity and mutualistic dependence strongly decreased the importance of empirical network structure for stability, the opposite was not true. The observed relationship reduced coextinction in empirical networks by 88% (figure 3*a*, empirical scenario) and by 86% in randomized networks (figure 3*a*, randomized scenario). We found qualitatively consistent patterns when using a broader set of relationships and relaxing the assumption that all species are obligate mutualists (electronic supplementary material, figure S3). Thus the positive relationship between partner diversity and mutualistic dependence observed empirically reduced the extent to which network structure minimized coextinction, conferred stability independent of network structure, and reversed predictions for the species most vulnerable to extinction.

## 4. Discussion

Using global databases and detailed field experiments from 30 seed dispersal networks, we found consistent support for a positive relationship between partner diversity and mutualistic dependence for both animals and plants in the seed dispersal mutualism. This empirical pattern caused an order-of-magnitude reduction in coextinction relative to predictions made using the typical assumption of network models that all species are strongly dependent on mutualistic interactions [7,10,11,13,24–27,35]. The causes of this reduction are twofold: (i) species' mutualistic strategies balance the risk of losing mutualistic interactions with their dependence on those mutualistic interactions, and (ii) these dynamics result in much lower dependence on mutualistic strategies than previously assumed. Species with high mutualistic dependence are buffered from coextinction by interacting with many partners, whereas species with low mutualistic dependence are buffered by their non-mutualistic alternatives for reproduction and survival. By focusing on the knowledge gap between network interaction data and the functional outcomes of these interactions, this work reveals ecological dynamics that cause mutualistic networks to be far more robust to coextinction than previously thought.

Mutualistic networks typically have a nested network structure where species with many partners interact both with other species that have many partners and also with species that have few partners, whereas species with few partners do not interact with others that have few partners [36]. Networks are thought to possess this structure in nature because it confers stability to mutualistic systems [13]; species considered most vulnerable to coextinction—those with few partners—decrease their vulnerability by interacting with species considered least vulnerable—those with many partners [8,9]. Our results suggest that this explanation for the emergence of nestedness does not hold after taking variation in species mutualistic strategy into account. Rather, we find that vulnerability to coextinction is similar across species with low and high partner diversity. When a species with a single partner is equally likely to lose a mutualist with high partner diversity as one with low partner diversity, a nested network structure would not reduce coextinction over a random network structure. Indeed, we found that nestedness did not strongly confer stability in our simulations after incorporating the empirical relationship between partner diversity and mutualistic dependence. Why would networks possess this structure if it does not strongly contribute to stability? Although there are other explanations that suggest nested structure derives from heterogeneity in species abundance, temporal or morphological constraints, or even sampling artefacts [37], the explanation we propose is rooted in the evolutionary ecology of mutualistic strategies.

A trade-off between mutualistic and non-mutualistic strategies should result in nested networks where partner diversity and mutualistic dependence are positively related. Species with high mutualistic dependence should invest heavily in maintaining mutualistic interactions and interact with many partners to ensure consistent mutualistic rewards or services. Species with low mutualistic dependence should invest little in maintaining mutualistic interactions and interact only with the partners that offer low-cost services or high-benefit rewards; importantly, these partners are species that invest heavily in maintaining mutualistic interactions. A strategy of high mutualistic dependence but low partner diversity should be selected against because it is too risky [16], and a strategy of low mutualistic dependence but high partner diversity should be selected against because it is too costly [21]. Therefore, a trade-off between mutualistic and non-mutualistic strategies—causing niche-based preferential attachment—would result in nestedness. Such a trade-off also explains the existence of species with few mutualists, which in previous network models were considered highly vulnerable to perturbation and thus did not appear to represent evolutionarily stable strategies. It also accounts for the lack of fully connected networks (each plant interacts with all animals and vice versa), a scenario that maximizes stability in topological (e.g. [7]), stochastic (e.g. [14]), and dynamical (e.g. [11]) network models, but which is unobserved in nature [36]. The trade-off explains the widespread pattern of nestedness, empirical patterns not explained by existing network models, and novel empirical patterns involving the functional outcomes of mutualistic interactions [18,20,22], such as the relationship between partner diversity and mutualistic dependence presented here.

Traits that influence the number of mutualists and investment in mutualistic strategy are likely to mediate a trade-off between mutualistic and non-mutualistic strategies, and may be useful predictors of mutualistic dependence. Highly frugivorous species (i.e. those with high mutualistic dependence) possess morphological adaptations such as large gapes and specialized gut morphology that allow them to process a wide range of fruit [38]. The benefits of these traits are likely to trade off with the frugivore's capacity to employ non-mutualistic strategies such as aerial insectivory or scavenging. A wide range of frugivores, including opportunistic frugivores, can handle small-seeded species [38] and may consume fruits that offer relatively greater nutritional rewards (figure 2*c*). However, the benefits of these traits for plants are likely to trade off with their ability to reproduce without animal mutualists. Compared to large-seeded plants that can be dispersed by fewer species, small-seeded species are less tolerant of the stressful conditions that non-dispersed seeds typically experience [39–41]. Compared to seeds that have little or no pulp and often employ non-mutualistic dispersal strategies (e.g. gravity or wind dispersal), non-dispersed seeds from fleshy fruits have reduced survival because remaining pulp can inhibit germination, attract predators, and facilitate pathogens [42,43]. Species traits have previously been used to gain insight into coextinction risk, with major emphasis on traits as predictors of partner diversity. In particular, large-seeded plant species are thought to be at highest coextinction risk because they have few potential large-bodied dispersers [44–46]. Our framework also suggests how traits of species can be employed to predict species’ mutualistic dependence, which is also critical for predicting the outcomes of mutualism disruption.

Our findings call for a re-evaluation of key conclusions derived from network models regarding the importance of network structure for coexistence and the importance of partner diversity for coextinction risk. After we incorporate the empirically derived relationship between partner diversity and mutualistic dependence into network predictions, we find that empirical network structure does relatively little to reduce coextinction (figure 3*a*), suggesting that the contribution of network structure for species coexistence is much smaller than previously reported [7,10,11,13]. Further, we find that species with few partners—often called ‘specialists’ in network studies—are not more vulnerable to coextinction, a model prediction that has been widely reported [10–13,26,28,35] and acknowledged as critical for the link between network structure and stability [28]. Rather than ‘specialists’ at high risk of losing their few partners [13], we find that species with few partners typically have generalized strategies involving low dependence on mutualistic interactions. Indeed, species that have specialized interactions and are obligate mutualists are extremely rare in nature [47], even in pollination systems that are typically more specialized [48]. These insights advance realistic predictions for the influence of network disruption on decline and extinction of individual mutualists. Models that incorporate empirical variation in mutualistic dependence or that include other interaction types in ‘multilayer’ networks [49,50] show strong potential for advancing our basic understanding of mutualistic network dynamics and for effectively applying network concepts to biodiversity conservation problems.

By pairing data on mutualistic interactions and their functional outcomes, we have shown that traits other than commonly studied network metrics are critical for understanding the sources of network stability and for our ability to predict coextinction. This reinforces the call for increased efforts to collect and synthesize data on the functional outcomes of mutualistic interactions in order to make strong ecological inferences and global change predictions [6]. A wide gap exists between network studies (typically using simple interaction data on many species) and field studies of mutualism (using detailed experiments to assess the influence of mutualism on individual vital rates of one or a few species). The difficulty of obtaining data on the functional outcomes of mutualistic interactions explains the absence of such empirical data in network research [51]. To achieve the significant potential of the network approach, ecologists must work to include empirical data of appropriate detail to balance the goal of ecological realism with the feasibility of obtaining data for many species within complex ecological communities [6].

Ecosystems around the world are experiencing unprecedented rates of species loss [52]. Time lags and extinction debts [53] are often suggested as explanations for the low number of coextinctions that have been found after contemporary mutualist loss [3]. Our findings offer an alternate explanation. The protection of obligate mutualists afforded by their connections to many partners, combined with the resistance of partial and opportunistic mutualists to the demographic impacts of partner extinction, makes mutualistic networks far more robust to coextinction than previously thought. These dynamics suggest the existence of late-stage tipping points [54] in network disassembly. In severely degraded networks, including those in increasingly common defaunated ecosystems [52] or those with multiple stressors [5], species that previously had many partners will face rapid declines due to their heavy dependence on the mutualism. Although this nonlinear response should reduce the resilience of mutualistic networks after anthropogenic stressors cause severe interaction loss, the same dynamics should make mutualistic networks far more resilient to initial species loss, creating a larger window of opportunity for conservation action.

## Supplementary Material

Supplementary methods, figures, and tables

## Data accessibility

Data and code are archived at the Dryad Digital Repository at http://dx.doi.org/10.5061/dryad.r1478 [55].

## References

[1] Barnosky AD et al. 2011 Has the Earth's sixth mass extinction already arrived? Nature 471, 51–57. (10.1038/nature09678) 21368823

[2] Janzen DH 1974 The deflowering of Central America. La deforestación de Centroamérica. Nat. Hist. 83, 48–53.

[3] Dunn RR, Harris NC, Colwell RK, Koh LP, Sodhi NS 2009 The sixth mass coextinction: are most endangered species parasites and mutualists? Proc. R. Soc. B 276, 3037–3045. (10.1098/rspb.2009.0413) PMC2817118 19474041

[4] Aslan CE, Zavaleta ES, Tershy B, Croll D 2013 Mutualism disruption threatens global plant biodiversity: a systematic review. PLoS ONE 8, e66993 (10.1371/journal.pone.0066993.s003) 23840571 PMC3686776

[5] Brodie JF, Aslan CE, Rogers HS, Redford KH, Maron JL, Bronstein JL, Groves CR 2014 Secondary extinctions of biodiversity. Trends Ecol. Evol. 29, 664–672. (10.1016/j.tree.2014.09.012) 25445878

[6] Howe HF 2016 Making dispersal syndromes and networks useful in tropical conservation and restoration. Glob. Ecol. Conserv. 6, 152–178. (10.1016/j.gecco.2016.03.002)

[7] Memmott J, Waser NM, Price MV 2004 Tolerance of pollination networks to species extinctions. Proc. R. Soc. Lond. B 271, 2605–2611. (10.1098/rspb.2004.2909) PMC1691904 15615687

[8] Bascompte J, Jordano P, Olesen JM 2006 Asymmetric coevolutionary networks facilitate biodiversity maintenance. Science 312, 431–433. (10.1126/science.1123412) 16627742

[9] Tylianakis JM, Laliberté E, Nielsen A, Bascompte J 2010 Conservation of species interaction networks. Biol. Conserv. 143, 2270–2279. (10.1016/j.biocon.2009.12.004)

[10] Fortuna MA, Bascompte J 2006 Habitat loss and the structure of plant–animal mutualistic networks. Ecol. Lett. 9, 281–286. (10.1111/j.1461-0248.2005.00868.x) 16958893

[11] Bastolla U, Fortuna MA, Pascual-García A, Ferrera A, Luque B, Bascompte J 2009 The architecture of mutualistic networks minimizes competition and increases biodiversity. Nature 458, 1018–1020. (10.1038/nature07950) 19396144

[12] James A, Pitchford JW, Plank MJ 2012 Disentangling nestedness from models of ecological complexity. Nature 487, 227–230. (10.1038/nature11214) 22722863

[13] Rohr RP, Saavedra S, Bascompte J 2014 On the structural stability of mutualistic systems. Science 345, 1253497 (10.1126/science.1253497) 25061214

[14] Vieira MC, Almeida Neto M 2015 A simple stochastic model for complex coextinctions in mutualistic networks: robustness decreases with connectance. Ecol. Lett. 18, 144–152. (10.1111/ele.12394) 25431016

[15] Jordano P 1987 Patterns of mutualistic interactions in pollination and seed dispersal: connectance, dependence asymmetries, and coevolution. Am. Nat. 129, 657–677. (10.1086/284665)

[16] Bond WJ 1994 Do mutualisms matter? Assessing the impact of pollinator and disperser disruption on plant extinction. Phil. Trans. R. Soc. Lond. B 344, 83–90. (10.1098/rstb.1994.0055)

[17] Blüthgen N, Fründ J, Vázquez DP, Menzel F 2008 What do interaction network metrics tell us about specialization and biological traits. Ecology 89, 3387–3399. (10.1890/07-2121.1) 19137945

[18] Tur C, Castro Urgal R, Traveset A 2013 Linking plant specialization to dependence in interactions for seed set in pollination networks. PLoS ONE 8, e78294 (10.1371/journal.pone.0078294) 24205187 PMC3813576

[19] Schleuning M et al. 2014 Ecological, historical and evolutionary determinants of modularity in weighted seed dispersal networks. Ecol. Lett. 17, 454–463. (10.1111/ele.12245) 24467289

[20] Mello M, Rodrigues FA, Costa LF, Kissling WD 2015 Keystone species in seed dispersal networks are mainly determined by dietary specialization. Oikos 124, 1031–1039. (10.1111/oik.01613)

[21] Bronstein JL 2001 The costs of mutualism. Am. Zool. 41, 825–839. (10.1093/icb/41.4.825)

[22] Schleuning M, Blüthgen N, Flörchinger M, Braun J, Schaefer HM, Böhning-Gaese K 2011 Specialization and interaction strength in a tropical plant–frugivore network differ among forest strata. Ecology 92, 26–36. (10.1890/09-1842.1) 21560673

[23] Gao J, Barzel B, Barabási A 2016 Universal resilience patterns in complex networks. Nature 530 307–312. (10.1038/nature16948) 26887493

[24] Rezende EL, Lavabre JE, Guimarães PR, Jordano P, Bascompte J 2007 Non-random coextinctions in phylogenetically structured mutualistic networks. Nature 448, 925–928. (10.1038/nature05956) 17713534

[25] Kaiser-Bunbury CN, Muff S, Memmott J, Müller CB, Caflisch A 2010 The robustness of pollination networks to the loss of species and interactions: a quantitative approach incorporating pollinator behaviour. Ecol. Lett. 13, 442–452. (10.1111/j.1461-0248.2009.01437.x) 20100244

[26] Thebault E, Fontaine C 2010 Stability of ecological communities and the architecture of mutualistic and trophic networks. Science 329, 853–856. (10.1126/science.1188321) 20705861

[27] Pocock MJO, Evans DM, Memmott J 2012 The robustness and restoration of a network of ecological networks. Science 335, 973–977. (10.1126/science.1214915) 22363009

[28] Saavedra S, Stouffer DB 2013 ‘Disentangling nestedness’ disentangled. Nature 500, E1–E2. (10.1038/nature12380) 23969464

[29] Bascompte J, Stouffer DB 2009 The assembly and disassembly of ecological networks. Phil. Trans. R. Soc. B 364, 1781–1787. (10.1098/rstb.2008.0226) 19451127 PMC2685423

[30] Wilman H, Belmaker J, Simpson J, la Rosa de C, Rivadeneira MM, Jetz W 2014 EltonTraits 1.0: species-level foraging attributes of the world's birds and mammals. Ecology 95, 2027 (10.1890/13-1917.1)

[31] Fricke EC, Tewksbury JJ, Rogers HS 2014 Multiple natural enemies cause distance-dependent mortality at the seed-to-seedling transition. Ecol. Lett. 17, 593–598. (10.1111/ele.12261) 24589220

[32] Rogers HS, Buhle ER, HilleRisLambers J, Fricke EC, Miller RH, Tewksbury JJ 2017 Effects of an invasive predator cascade to plants via mutualism disruption. Nat. Commun. 8, 14557 (10.1038/ncomms14557) 28270682 PMC5344968

[33] Vazquez DP, Ramos Jiliberto R, Urbani P, Valdovinos FS 2015 A conceptual framework for studying the strength of plant–animal mutualistic interactions. Ecol. Lett. 18, 385–400. (10.1111/ele.12411) 25735791

[34] Patefield WM 1981 Algorithm AS 159: an efficient method of generating random R×C tables with given row and column totals. Appl. Stat. 30, 91 (10.2307/2346669)

[35] Saavedra S, Stouffer DB, Uzzi B, Bascompte J 2011 Strong contributors to network persistence are the most vulnerable to extinction. Nature 478, 233–235. (10.1038/nature10433) 21918515

[36] Bascompte J, Jordano P, Melian CJ, Olesen JM 2003 The nested assembly of plant–animal mutualistic networks. Proc. Natl Acad. Sci. USA 100, 9383–9387. (10.1073/pnas.1633576100) 12881488 PMC170927

[37] Vazquez DP, Bluthgen N, Cagnolo L, Chacoff NP 2009 Uniting pattern and process in plant-animal mutualistic networks: a review. Ann. Bot. 103, 1445–1457. (10.1093/aob/mcp057) 19304996 PMC2701748

[38] Jordano P 2000 Fruits and frugivory. In Seeds: the ecology of regeneration in natural plant communities (ed. Fenner M), pp. 125–166, 2nd edn Wallingford, UK: CABI.

[39] Tilman D 1994 Competition and biodiversity in spatially structured habitats. Ecology 75, 2–16. (10.2307/1939377)

[40] Muller-Landau HC 2010 The tolerance–fecundity trade-off and the maintenance of diversity in seed size. Proc. Natl Acad. Sci. USA 107, 4242–4247. (10.1073/pnas.0911637107) 20160078 PMC2840174

[41] Lebrija-Trejos E, Reich PB, Hernandéz A, Wright SJ 2016 Species with greater seed mass are more tolerant of conspecific neighbours: a key driver of early survival and future abundances in a tropical forest. Ecol. Lett. 19, 1071–1080. (10.1111/ele.12643) 27346439

[42] Janzen DH 1977 Why fruits rot, seeds mold, and meat spoils. Am. Nat. 111, 691–713. (10.1086/283200)

[43] Traveset A, Robertson A, Rodríguez-Pérez J 2007 A review on the role of endozoochory on seed germination. In Seed dispersal: theory and its application in a changing world, pp. 78–103. Wallingford, UK: CABI Publishing.

[44] Nuñez-Iturri G, Olsson O, Howe HF 2008 Hunting reduces recruitment of primate-dispersed trees in Amazonian Peru. Biol. Conserv. 141, 1536–1546. (10.1016/j.biocon.2008.03.020)

[45] Terborgh J, Nuñez-Iturri G, Pitman NCA, Valverde FH. C, Alvarez P, Swamy V, Pringle EG, Paine CET 2008 Tree recruitment in an empty forest. Ecology 89, 1757–1768. (10.1890/07-0479.1) 18589539

[46] Kurten EL, Wright SJ, Carson WP 2015 Hunting alters seedling functional trait composition in a Neotropical forest. Ecology 96, 1923–1932. (10.1890/14-1735.1) 26378314

[47] Bluthgen N, Menzel F, Hovestadt T, Fiala B, Blüthgen N 2007 Specialization, constraints, and conflicting interests in mutualistic networks. Curr. Biol. 17, 341–346. (10.1016/j.cub.2006.12.039) 17275300

[48] Waser NM, Ollerton J 2006 Plant–pollinator interactions: from specialization to generalization. Chicago, IL: The University of Chicago press.

[49] Boccaletti S, Bianconi G, Criado R, del Genio CI, Gómez-Gardeñes J, Romance M, Sendiña-Nadal I, Wang Z, Zanin M 2014 The structure and dynamics of multilayer networks. Phys. Rep. 544, 1–122. (10.1016/j.physrep.2014.07.001) PMC7332224 32834429

[50] Genrich CM, Mello MAR, Silveira FA. O, Bronstein JL, Paglia AP 2016 Duality of interaction outcomes in a plant–frugivore multilayer network. Oikos 126, 361–368. (10.1111/oik.03825)

[51] Schleuning M, Fründ J, Garcia D 2015 Predicting ecosystem functions from biodiversity and mutualistic networks: an extension of trait based concepts to plant–animal interactions. Ecography 38, 380–392. (10.1111/ecog.00983)

[52] Dirzo R, Young HS, Galetti M, Ceballos G, Isaac NJB, Collen B 2014 Defaunation in the Anthropocene. Science 345, 401–406. (10.1126/science.1251817) 25061202

[53] Tilman D, May RM, Lehman CL, Nowak MA 1994 Habitat destruction and the extinction debt. Nature 371, 65–66. (10.1038/371065a0)

[54] Barnosky AD et al. 2012 Approaching a state shift in Earth's biosphere. Nature 486, 52–58. (10.1038/nature11018) 22678279

[55] Fricke EC, Tewksbury JJ, Wandrag EM, Rogers HS 2017 Mutualistic strategies minimize coextinction in plant–disperser networks. *Dryad Digital Repository*. (10.5061/dryad.r1478) PMC5443928 28490622

