# Supplementary material for: Mutualistic strategies minimize coextinction in plant–disperser networks
Source: Proc Biol Sci. 2017 May 10;284(1854):20162302. doi: 10.1098/rspb.2016.2302 (PMC5443928; doi:10.1098/rspb.2016.2302)
Supplement: Supplementary methods, figures, and tables [file rspb20162302supp1.pdf]

Electronic supplementary material

Appendix S1

Seed dispersal interactions and benefits of seed dispersal for plants in the Mariana Islands. Our observations and experiments were conducted in native limestone karst forest, the primary forest type across the islands. Birds are the primary seed dispersing species in this system. The introduced emerald tree skink (*Lamprolepis smaragdina*) and green anole (*Anolis carolinensis*) occasionally consume fruit during the day as well. The only other likely frugivore is a single fruit bat species (*Pteropus mariannus*), which is rare across these islands. Its extremely low population densities on Saipan suggest the absence of a functional role in seed dispersal [56]. We therefore limited our analysis of the seed dispersal mutualism to diurnal interactions.

Seed dispersal network. We recorded frugivory through extended direct observation of focal fruiting trees [22]. Observers surveyed study sites for representative fruiting trees and rotated among sites and available tree species. Using binoculars, observers recorded the number of diaspores manipulated, distinguishing between interactions where the diaspore was consumed, those where the diaspore was dropped, or those where pulp was removed without dispersal. We analyse only data on the number of diaspores consumed. Our analysis covered six tree species, *Aglaia mariannensis* (Meliaceae), *Carica papaya* (Caricaceae), *Melanolepis multiglandulosa* (Euphorbiaceae), *Pipturus argenteus* (Urticaceae), *Premna serratifolia* (Lamiaceae), and *Psychotria mariana* (Rubiaceae), hereafter referred to by genus. These species are those that we observed during both years and whose seeds we observed to be dispersed by vertebrates.

For one species, *Carica*, we used video recordings as well as direct observations to record frugivory events. Species recorded consuming seeds were the golden white-eye (*Cleptornis marchei*), bridled white-eye (*Zosterops conspicillatus*), Micronesian starling (*Aplonis opaca*), white-throated ground dove (*Gallicolumba xanthonura*), Mariana fruit dove (*Ptilinopus roseicapilla*), emerald tree skink (*Lamprolepis smaragdina*), and green anole (*Anolis carolinensis*). Observations were conducted between 6:00 AM and 2:00 PM. We recorded data on an average of 23 individuals per species (range 13-43 individuals). Observation periods lasted between 2 and 8 hours and the total duration of observation per species averaged 220 hours (range 102-386 hours). During each observation period, we recorded the number of ripe fruits visible on each focal tree. For species with multiple diaspores per fruit we multiplied the number of visible fruits by the mean number of diaspores per fruit to estimate number of ripe diaspores. We standardize interaction frequency as the per-capita frugivory rate (portion of ripe diaspores consumed per hour) and calculate species strength, species degree, the total interaction frequency, and Shannon diversity.

Distance- and light-dependent survival: field seed additions. We measured survival at the seed to seedling transition for *Aglaia* and *Premna* using seed additions near and far from conspecific adults on Saipan. These experiments are described in detail in Fricke et al. [31]. We added seeds in circular 0.05 m^2 plots, with six near and six far plots per species at each of three forest sites. Seeds were collected from at least 10 individuals and were sown at densities of 20 seeds per plot for *Aglaia* and 50 seeds per plot for *Premna*. Because these plots were control plots used in experiments testing natural enemy impacts on survival, they were partially surrounded by wire mesh that

allowed for a consistent light environment with the other experimental plots but that did not influence natural enemy access. We measured survival of seedlings 5 weeks after germination began for *Premna* and 10 weeks after germination began for *Aglaia*. We recorded canopy openness above each plot using a spherical densiometer.

Distance- and light-dependent survival: field seedling additions. We measured survival at the seedling stage for *Aglaia*, *Carica*, *Psychotria*, and *Premna* using seedling additions near and far from conspecifics on Saipan, and the nearby Mariana Islands of Tinian, Rota, and Guam, experiments described in detail in Rogers et al. [32]. We transplanted seedlings grown in shadehouses into circular ~1.2 m^2 plots, with four near (under canopy) and four far (typically > 7m away from conspecifics) plots per species at each forest site where conspecifics were present. Saipan, Tinian, and Rota each had three forest sites and Guam had five forest sites. We planted seedlings at a density of 10 per plot. We controlled for any effect of introduced ungulates that are present on Guam and Rota using 1 m tall poultry netting fences, and used fences around plots on all islands. We measured seedling survival after 4-7 months, depending on species identity and island. We recorded canopy openness above each plot using a spherical densitometer.

Distance-dependent survival: shade-house experiments. We compared survival over the seed to seedling transition for *Premna* and *Psychotria* seeds grown with soil taken either under the canopy of adult conspecifics or 30 m away from the nearest conspecific. Soil for each treatment was collected from at least three locations, mixed thoroughly, and passed through a 1 mm mesh that removed any conspecific seeds. Each seed was sown in individual cells containing one part field soil and three parts of a 1:1 perlite and peat moss mix. Each species by treatment combination had a minimum of 274

seeds. We measured seedling survival 10 weeks after sowing seeds, although some viable seeds would likely have germinated later. Germination began approximately one week after seeds were sown for *Premna* and approximately 6 weeks for *Psychotria*.

Frugivore handling: shade-house experiments. We compared germination of *Premna* and *Psychotria* seeds that were either handled or not handled by frugivores in a shadehouse experiment. Seed traps were placed under fruiting trees on Saipan, Tinian and Rota to collect falling fruit and seeds. Seeds that did not have pulp remaining on the seed were considered handled. We planted whole fruits and handled seeds in individually labelled cells. Each species by treatment combination had a minimum of 117 seeds. Plants were watered and checked for germination daily for six months, but no new germinants were recorded after four months and we assume all viable seeds had germinated.

Gap survival: field seed additions. We compared survival in natural canopy gaps and in nearby closed-canopy areas for *Aglaia*, *Carica*, and *Premna* using seed additions. We performed these experiments on Guam because canopy gaps are more prevalent on Guam than the other islands, allowing sufficient replication, and because germination from background seed rain away from conspecifics is likely minimal due to the functional extirpation of frugivorous birds on Guam. We systematically searched the area surrounding our mapped forest sites for canopy gaps that were 5-10 m^2 in area, the gap size typical of canopy gaps in the Marianas. Four forest sites had three or more suitable gaps, and we randomly selected three as experimental plots. To obtain a paired closed-canopy plot for each gap plot, we followed a random compass bearing 10 m away from the edge of the canopy gap, and established a closed-canopy plot if the substrate of the

potential closed-canopy plot was qualitatively similar to that in the gap plot. If not, the process was repeated with another random compass bearing. Each ~7 m^2 plot was fenced to exclude non-native ungulates. Seeds were sown into 0.25 m^2 plots at a density of 75 per plot for *Aglaia*, 125 per plot for *Carica*, and 150 per plot for *Premna* to approximately scale densities inversely with seed size. We recorded the number of germinants in each plot weekly for 20 weeks.

Dispersal benefit analyses. To quantify the benefit of seed dispersal, we calculated a 'dispersal benefit ratio' that is the ratio of survival for seeds or seedlings in a 'dispersed' scenario (far from conspecifics, in canopy gaps, etc.) versus in a 'non-dispersed' scenario (near conspecifics, under closed canopy, etc.). We fitted generalized linear mixed effects models with binomial errors using the R package MCMCglmm [57]. This approach allowed us to obtain estimates and credible intervals for the ratio of certain levels of categorical variables or quantiles of continuous variables, which represent the dispersed and non-dispersed scenarios. All analyses used uninformative inverse Gamma priors for the residual variance, setting the mean of the inverse Wishart distribution to 1 and the degree of belief parameter to 0.002. We fix unit-level variance to 1 only in models with a Bernoulli response variable (distance dependence and handling shadehouse experiments) because unit-level variances cannot be estimated in those cases. We used parameter expanded priors for the random effects with mean 0 and variance 1000. For all models, we performed 300,000 iterations, used a thinning interval of 50, and discarded the first 10,000 iterations as burn-in. For distance-dependent seedling mortality, the response variable was the portion of seedlings surviving out of the seedlings planted. The fixed effects were distance (near or far) and canopy openness. The random effects were

site and island. For distance-dependent mortality at the seed to seedling transition, the response variable was the portion of seedlings surviving out of seeds sown. The fixed effects were distance and canopy openness. The random effects were nearest grid point within each mapped forest site. For shadehouse distance-dependent survival, the response variable was survival as a Bernoulli response. The fixed effect was soil type (near or far). The random effect was the parent tree. For shadehouse frugivore handling, the response variable was germination as a Bernoulli response. The fixed effect was handling treatment (handled or unhandled). The random effects were site and island. For gap-dependent survival, the response variable was the portion seeds sown that germinated. The fixed effect was light environment (gap or closed canopy). The random effects described pairs of gap and closed-canopy plots. To obtain species-specific estimates, all models had interactions by species with each of the fixed effects. We considered dispersed versus non-dispersed scenarios as far from conspecifics versus near conspecifics, in canopy gap versus under closed canopy, handled by frugivores versus not handled, and in high light microsites representing the 90th percentile of recorded canopy openness values versus the 10th percentile of canopy openness. The dispersal benefit ratio was calculated as the mean log-transformed ratio of posterior estimates of survival from each of the dispersed vs. non-dispersed scenarios.

To assess relationships between partner diversity and the benefits of dispersal, we used a linear mixed effects model with the mean dispersal benefit ratio as the dependent variable and species strength, species degree, the total interaction frequency, or Shannon diversity as the fixed effect in separate models. Treating each of the 7 benefit type-by-species stage combinations (distance dependence at the seed stage, distance dependence

at the seedling stage, etc.) as a random effect, we allowed random slopes and intercepts in the lme4 package [58]. To determine statistical significance, we used likelihood ratio tests to compare the models described above to intercept-only models lacking the fixed effect. The presence of a positive effect of species strength would indicate that, even with variation in the magnitude of the benefit or the slope of its relationship, species with greater species strength have greater benefits of dispersal.

Seed and fruit measurements. We measured pulp-to-seed ratios from fruit collected on Guam. We collected ripe fruits from multiple individuals at multiple forest sites. A minimum of 24 diaspores per species were used to obtained seed and pulp data. Pulp was considered fruit tissue excluding seeds and any tissue that frugivores were observed not to consume. Seed and pulp samples were dried at 65C to a constant mass. We report the mean pulp-to-seed ratio calculated as mean pulp mass per diaspore / mean seed mass per diaspore.

Additional coextinction predictions. *Coextinction scenarios:* In addition to simulations constrained by the empirical relationship between partner diversity and mutualistic dependence, we performed additional sets of coextinction simulations to assess how variation in the strength of the positive relationship between partner diversity and mutualistic dependence and empirical network structure each influences coextinction predictions. Using the 11 quantitative seed dispersal networks, we predicted the number of coextinctions under six scenarios.

In the "obligate" scenarios, $R_i = 1$ for all species. In the "positive relationship" scenarios, there was a linear relationship between species strength and mutualistic dependence. For consistency across networks and ease of interpretation, we calculated

relative species strength that varies between 0 and 1 by dividing the species strength of plants and animals by the species strength of the plant and animal species with the highest species strength present in the network. To vary the strength of the positive relationship, we varied its slope while bounding R_i to 1 when relative species strength equals 1. In other words, a slope of 0 indicates that $R_i = 1$ for all species (and corresponds to an assumption that all species are obligate mutualists); a slope of 0.6 indicates that mutualistic dependence equals 0.4 when relative species strength equals 0 and 1 when relative species strength equals 1. We considered slope values between 0 and 1. The "positive null" scenarios were used to correct for the overall decrease in mutualistic dependence that larger slope values necessarily create, and are thus used to isolate the influence of the positive relationship between species strength and mutualistic dependence from the influence of a system-wide decrease in mutualistic dependence. To do so, the "positive null" scenarios instead used a horizontal relationship, indicating partial mutualists to varying degrees. For each slope value tested, the area under the horizontal relationship used for the "positive null" scenario was equal to the area under the positive relationship used for the "positive relationship" scenario.

For each of the "obligate", "positive relationship", and "positive null" scenarios, we performed simulations using either empirical networks or randomized networks. In each iteration of the simulation, coextinctions were predicted for each scenario using the same randomly chosen species to initiate the coextinction cascade and, for the randomized network scenarios, the same randomized network. The number of resulting coextinctions was recorded in each of the six scenarios. For each network, we performed 200 iterations of the simulation at each of 100 slope values between 0 and 1. For data

visualization of the relative difference in coextinction between two scenarios, we took the ratio of the number of coextinctions averaged across the 200 iterations between the two scenarios at each slope value, fitting a spline as a fit line. For data visualization of the portion of species experiencing coextinction, we used a generalized linear mixed effects model with the number of coextinctions out of the number of species in the network as the response variable, the slope value as the predictor variable, random slopes and intercepts by network ID, and the binomial error distribution in the lme4 package (Bates et al. 2016).

Electronic supplementary materials references

56. McConkey, K. R. & Drake, D. R. 2006 Flying foxes cease to function as seed dispersers long before they become rare. *Ecology* **87**, 271–276.

57. Hadfield, J. D. 2010 MCMC methods for multi-response generalized linear mixed models: the MCMCglmm R package. *Journal of Statistical Software*

58. Bates, D., Mächler, M., Ben Bolker & Walker, S. 2015 Fitting Linear Mixed-Effects Models Using lme4. *Journal of Statistical Software* **67**, 1–48. (doi:10.18637/jss.v067.i01)

Electronic supplementary materials figures and tables

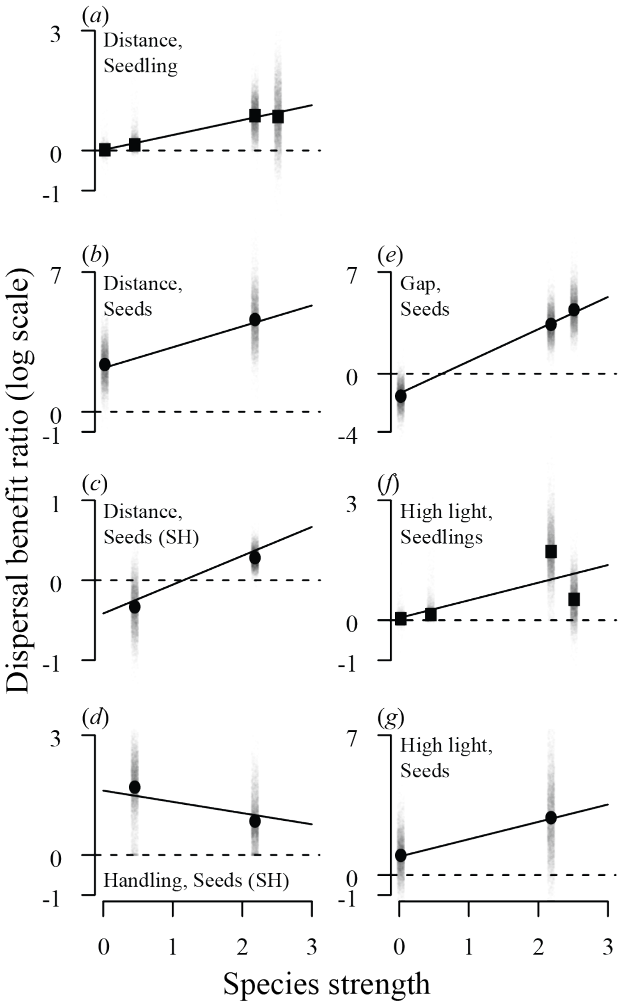

Figure S1. Benefits of seed dispersal increase with species strength for trees in the Mariana Islands. Figures represent model outputs of the dispersal benefit ratio (ratio of survival of dispersed seeds or seedlings to survival of non-dispersed seeds or seedlings) assessed in experiments measuring benefits associated with movement away from adult conspecifics (Distance; *a-c*), handling of seeds by frugivores (Handling; *d*), and movement to canopy gaps (Gap; *e*) or high light microsites (High light; *f-g*). Experiments were conducted by sowing seeds (Seeds; circles; *b-e,g*) or planting seedlings (Seedlings; squares; *a,f*). Experiments performed in a shadehouse are indicated with “SH”;

experiments were otherwise performed in the field. Grey bars show posterior estimates for the dispersal benefit ratio, points indicate the mean dispersal benefit ratio, and lines represent the model fit.

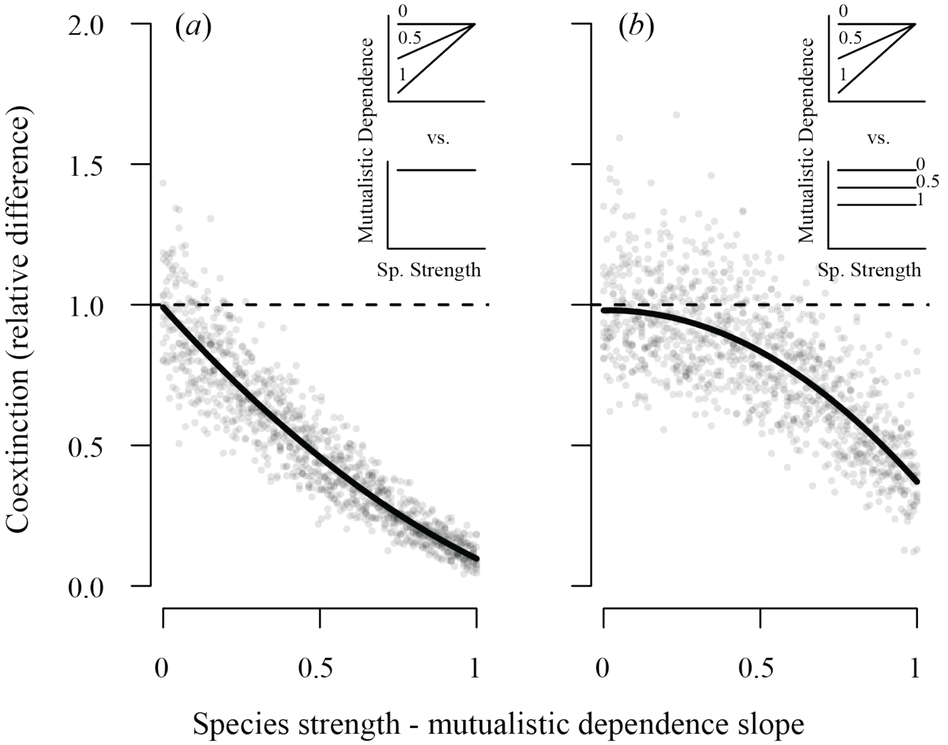

Figure S2. Impact on coextinction of positive relationships between partner diversity and mutualistic dependence. The relative difference in coextinction is presented as the ratio of coextinctions; the numerator is the number of coextinctions under the ‘positive relationship’ scenario (greater positive relationships between species strength and mutualistic dependence in the inset panels of *a* and *b*) and the denominator is the number of coextinctions predicted in either the ‘obligate’ scenario (bottom inset panel in *a*) or the ‘positive null’ scenario (bottom inset panel in *b*). For each species strength-mutualistic dependence slope value, the area under the horizontal ‘positive null’ relationship equals the area under the relationship used for the ‘positive relationship’ scenario. Increasingly

positive relationships between species strength and mutualistic dependence decrease coextinction (*a*), even when compared to the null model that accounts for the overall decreases in mutualistic dependence caused by positive species strength-mutualistic dependence relationships (*b*). Here the maximum value of mutualistic dependence is 1, but coextinction probabilities are further decreased when lower maximum values of mutualistic dependence are used in the simulation.

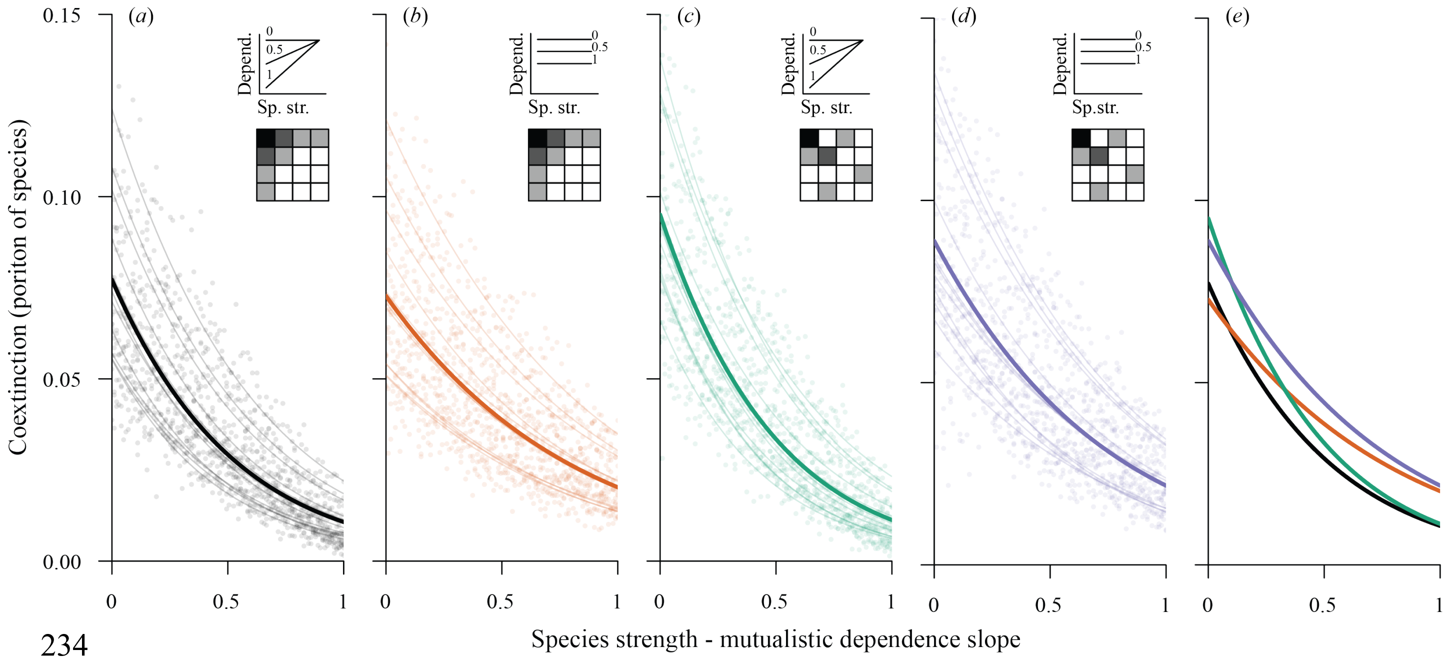

Figure S3. Comparing the impact on coextinction of empirical network structure and of a positive relationship between partner diversity and mutualistic dependence. Four scenarios (*a*-*d*) represent each combination of the presence or absence of a positive species strength-mutualistic dependence relationship and of empirical or randomized network structure. Inset line plots show how the relationship between species strength (Sp. Str.) and mutualistic dependence (Depend.) varies for different values of species strength-mutualistic dependence slope in either the ‘positive relationship’ (increasingly

positive relationships; *a,c*) or ‘positive null’ (horizontal relationships; *b,d*) scenarios. Inset bipartite plots show whether empirical (*a,b*) or randomized (*c,d*) networks were used in the simulations. Points represent the portion of species in a network experiencing extinction in a given iteration of the simulation. Thin lines are model fits for each of the 11 empirical networks and the thick line in each panel is the mean relationship across all networks, each of which is overlaid in *E*. The existence of a positive relationship decreases coextinction to a greater extent than a corresponding simple decrease in mutualistic dependence in both empirical (*a* vs. *b*) and randomized (*c* vs. *d*) networks. Relative to a randomized network structure, the empirical network structure decreases coextinction in the presence (*a* vs. *c*) or absence (*b* vs. *d*) of a positive relationship between species strength and mutualistic dependence. However, this benefit of empirical nested structure decreases (the relative difference between *a* and *c* becomes smaller) with more positive species strength-mutualistic dependence relationships.

Table S1. References and network description for 29 networks included in the analysis. Descriptors include a unique network identification from the Web of Life database (ID), number of plant and animal species in the network (Spp), total number of interactions (Int), connectance (Con), network type (Type; quantitative=1, binary=0), the reference, locality, latitude, and longitude.

ID	Spp	Int	Con	Type	Reference	Locality	Lat	Long
M_SD_001	28	50	0.34	1	Baird, J.W. 1980. The selection and use of fruit by birds in an eastern forest. Wilson Bulletin 92: 63-73.	Princeton, Mercer, New Jersey, USA	40.37	-74.67
M_SD_002	40	119	0.427	1	Beehler, B. 1983. Frugivory and polygamy in birds of paradise. Auk, 100: 1-12.	Mount Missim, Morobe Prov., New Guinea	-7.27	146.7
M_SD_003	41	68	0.17	1	Carlo et al. (2003) Avian fruit preferences across a Puerto Rican forested landscape: pattern consistency and implications for seed removal. Oecologia 134: 119-131	Caguana, Puerto Rico	18.30	-66.78
M_SD_004	54	95	0.14	1	Carlo et al. (2003) Avian fruit preferences across a Puerto Rican forested landscape: pattern consistency and implications for seed removal. Oecologia 134: 119-131	Cialitos, Puerto Rico	18.26	-66.54
M_SD_005	38	49	0.151	1	Carlo et al. (2003) Avian fruit preferences across a Puerto Rican forested landscape: pattern consistency and implications for seed removal. Oecologia 134: 119-131	Cordillera, Puerto Rico	18.17	-66.59
M_SD_006	36	51	0.162	1	Carlo et al. (2003) Avian fruit preferences across a Puerto Rican forested landscape: pattern consistency and implications for seed removal. Oecologia 134: 119-131	Fronton, Puerto Rico	18.31	-66.56
M_SD_007	79	143	0.284	0	Crome, F.H.J. 1975. The ecology of fruit pigeons in tropical Northern Queensland. Australian Journal of Wildlife Research, 2: 155-185.	Tropical rainforest. Queensland, Australia.	-17.85	146.08
M_SD_009	25	38	0.302	1	Galetti, M., Pizo, M.A. 1996. Fruit eating birds in a forest fragment in southeastern Brazil. Ararajuba, Revista Brasileira de Ornitologia, 4: 71-79.	Santa Genebra Reserve T1. SE Brazil	-22.82	-47.1
M_SD_010	64	234	0.334	1	Snow, B.K., Snow, D.W. 1971. The feeding ecology of tanagers and honeycreepers in Trinidad. Auk, 88: 291-322.	Tropical rainforest. Trinidad.	10.72	-61.3

M_SD_011	25	47	0.305	0	Snow B.K. & Snow D.W. (1988) Birds and berries, Calton, England.	Calton, Great Britain	53.06	-1.85
M_SD_012	64	146	0.144	1	Galetti, M., Pizo, M.A. 1996. Fruit eating birds in a forest fragment in southeastern Brazil. Ararajuba, Revista Brasileira de Ornitologia, 4: 71-79.	Santa Genebra Reserve T2. SE Brazil	-22.82	-47.1
M_SD_013	55	197	0.288	0	Hammann, A. & Curio, B. (1999) Interactions among frugivores and fleshy fruit trees in a Philippine submontane rainforest.	North Negros Forest Reserve, Central Philippi	10.68	123.18
M_SD_014	33	121	0.445	0	Jordano P. (1985) El ciclo anual de los paseriformes frugÃvoros en el matorral mediterrÃ¡neo del sur de EspaÃ±a: importancia de su invernada y variaciones interanuales. Ardeola, 32, 69-94.	Hato RatÃ³n, Sevilla. Spain	37.18	-6.32
M_SD_015	32	86	0.637	0	Kantak, G.E. 1979. Observations on some fruit-eating birds in Mexico. Auk, 96: 183-186.	Campeche state, Mexico	18.51	-89.49
M_SD_016	85	500	0.342	0	Lambert F. (1989). Fig-eating by birds in a Malaysian lowland rain forest. J. Trop. Ecol., 5, 401-412.	Kuala Lompat, Krau Game Reserve.	3.72	102.28
M_SD_017	24	72	0.562	0	Tutin, C.E.G., Ham, R.M., White, L.J.T., Harrison, M.J.S. 1997. The primate community of the LopÃ© Reserve, Gabon: diets, responses to fruit scarcity, and effects on biomass. American Journal of Primatology, 42: 1-24.	Gabon, Africa	0.17	11.58
M_SD_018	61	66	0.071	0	Mack, AL and Wright, DD. (1996) Notes on occurrence and feeding of birds at Crater Mountain Biological Research Station, Papua New Guinea. Emu 96: 89-101.	Crater Mountain Biological Research Station,	-6.72	145.09
M_SD_019	209	666	0.099	0	Wheelwright, N.T., Haber, W.A., Murray, K.G., Guindon, C. 1984. Tropical fruit-eating birds and their food plants: a survey of a Costa Rican lower montane forest. Biotropica, 16: 173-192.	Monteverde, Costa Rica	10.3	-84.8
M_SD_020	58	150	0.182	1	P. Jordano, unpubl.	Nava Correhuelas. S. Cazorla, SE Spain.	37.93	-2.87

M_SD _021	46	129	0.256	0	P. Jordano, unpubl.	Nava Noguera, Sierra de Cazorla, SE Spain	37.94	-2.79
M_SD _022	317	112 1	0.049	0	Silva, W.R., P. De Marco, E. Hasui, and V.S.M. Gomes (2002) Patterns of fruit-frugivores interactions in two Atlantic Forest bird communities of South-eastern Brazil: implications for conservation. Pp. 423-435. In: D.J. Levey, W.R. Silva and M. Galetti (eds.) Seed dispersal and frugivory: ecology, evolution and conservation. Wallinford: CAB International.	Intervales and Saibadela, São Paulo, Brazil	-24.27	-48.41
M_SD _023	23	38	0.317	1	Noma, N. 1997. Annual fluctuations of sapfruits production and synchronization within and inter species in a warm temperate forest on Yakushima Island, Japan. Tropics, 6: 441-449.	Yakushima Island, Japan	30.33	130.5
M_SD _024	19	40	0.476	0	Guitián, J. (1983). Relaciones entre los frutos y los passeriformes en un bosque montano de la cordillera cantábrica occidental. PhD Thesis, Univ. Santiago, Spain.	NW Spain temperte forest		
M_SD _025	13	22	0.524	0	Sorensen 1981	England	51.77	-1.33
M_SD _026	6	6	0.667	0	Data unpublished, however see Jordano 1993	Campos de Otero, Sierra Nevada	37.11	-3.39
M_SD _027	16	31	0.646	0	Data unpublished, however see Jordano 1993	Cañada de las Sabinas, Nava de las Correhuelas, Sierra de Cazorla	37.93	-2.87
M_SD _028	13	26	0.65	0	Data unpublished, however see Jordano 1993	Cañada Travina, Sierra de Cazorla	38.08	-2.75
M_SD _029	9	10	0.5	0	Data unpublished, however see Jordano 1993	Sabinar del Marqués, Reserva de la Estación Biológica de Doñana	37.02	-6.55
M_SD _030	9	11	0.55	0	Data unpublished, however see Jordano 1993	Santa Bárbara, Sierra de Baza	37.38	-2.84
